# Digitalis in the Treatment of Mania a Potu

**Published:** 1861-02

**Authors:** G. C. Catlett


### *Digitalis in the treatment of Mania a Potu.* By G. C. Catlett, M. D.

The experience of the Physicians of this city, so far as my knowledge extends in the treatmeat of Mania a Potu, has been very unsatisfactory. Most of the attacks assuming a remarkably acute form; with great violence of delirium, furious mania, and persistent mental aberrations, have generally resisted the opium and stimulating as well as all of the former establihed methods of treatment for this unfortunate class of sufferers.

So great has been the mortality from this disease during the last four or five years, uotwithstanding we have attempted to discriminate between the chronic and the acute forms; that we have become apprehensive that we have not been treating alcoholic poision. Indeed so general has become the impression that the various alcoholic drinks generally used, contain a foreign poison, that those who are in the habit of becoming intoxicated are significantly said to have taken passage on the strychnine line for an unknown destination. While the symptoms of the numerous cases that have come under my observation do not in the slightest resemble those from the poison of Strychnia, yet, the great apparent difference in the character of the symptoms as well as in the result of the disease—produced by excessive use of alcohol—at the present and former periods, that it would very naturally create apprehensive and strike terror to the spiritually infatuated.

There have been a few persons, by a prolonged debauch have induced inflammation of the mucous membrane of the stomach or at least, such a degree of irritability that a continuation of indulgence was impossible, as nothing introduced into it would be retained, soon therefore from the withdrawal of the stimulous, delirium tremens would supervene, characterized by intense hallucinations, great tremulousness, incapacity to sleep, irritability of stomach, tender epigastrium and deficiency of the secretions. Counter irritation to the epigastrium, a mercurial cathartic, stimulants judiciously administered (I prefer Tinct. Serpentaria; and Tinct. Valerian, equal proportions,) and Morphia *pro renata*, will generally in my hands relieve such cases. But that form of this disease that is produced by the constant daily use of alcohol long continued, poisoning the blood and resulting in acute mania, is the fatal form, or properly Mania a Potu.—In relation to this form of the disease Prof. Stone of New Orleans says:

"Brain fever and apoplexy are terms often kindly substituted as being more respectable; but names do not alter facts. Mania a potu usually occurs with the robust who habitually use acoholic stimulents, but not to any great excess, except upon occasions, and when they are carried to a certain extent, a necessity for their continuance is created, and their excessive use cannot, or will not, be resisted until the stomach gives way and finally rejects them. During this process the mucous membrane becomes engorged, the digestion, and finally the appetite, entirely fail, and the patient is sustained for some days after, by stimulants alone, until furious delirium sets in.

This madness is not due to the stoppage of an accustomed stimulant, for it often set in while the subject is in the full use of it, but it is plainly due to alcoholic poison and the abscence of proper nutritive matter in the blood. I think I may add another cause which has often something to do in causing the delirium, and certainly much to do in causing death, under some modes of treatment, and that is suppressed excretions. So long as the stomach is intact, and the apetite and digestion good, an immense quantity of stimulant may be disposod of without serious immediate consequences; but when the organs finally, from constant excitation, become engorged, nutrition ceases, and the alcohol is retained more in the blood, instead of being carried off by excretions, and a wild delirium soon follows.

It is plain under these circumstances, that the indicatious are to establish the excretions, disgorge the system of the alcoholic poison, and to introduce proper nutriment. The first two are accomplished by one and the same means. The stomach is generally irritable; at least there is frequent vomiting; but it is owing to the accumulation in the stomach of morbid secretion, rather than from inflammation or even irritation; for calomel in small doses frequently repeated, arrest it with great certainty. If the subject is governable, and will take medicine willingly, calomel should be given in two or three grain doses every hour, or oftener if the case is urgent, until fifteen or twenty grains are given: if medicine has to be given by force, it is best to give a full dose at once; and this is better, for in the worst, the stomach is not nauseated, and the sedative effect, of a large dose of calomel calms the nervous excitement and at the same time produces the appropriate effect upon the excretory organs and mucous coat of the stomach and bowels. It requires some hours for this effect to be produced, and it is improper to give anything to promote its action upon the bowels under ten or twelve hours, and I think even a longer time would be better, if the case is not urgent. Small and frequently repeated doses of saline medicine are the best, after the calomel (sulphate of magnesia is best), which promotes the excretions, disgorges the stomach and bowels, and clears the system of its alcoholic poison, to its great relief. An active cathartic may afford some relief, but the system is not so well disgorged by it; more or less serum from the blood is carried off, causing weakness; while, in the other process, by giving time for the action of the calomel, and then promoting it by gentle but continued means, the organs exercise a selection in excreting, and thereby a large amount of effete matter is discharged, and the patient feels the stronger for it, being freed from an incubus that was weighing it down, and producing apparent exhaustion. After this process, we should loose no time in introducing nutriment, and for this purpose milk is almost universally applicable; and as the mucous membrane of the stomach seems to be denuded of its epthelium, the addition of lime water renders it particularly grateful and soothing. Patients in this condition generally loathe animal substances, but milk is almost always grateful to the taste, and is particularly appropriate for it furnishes the most innocent solid for the bowels, that have been long deprived of their wholesome stimulus. If it should happen that a patient could not take milk, well boiled cornmeal gruel is the next best diet most likely to be relished; and for something more substantial, strong, well-seasoned bioth, frozen will be the most likely to agree.

In all acute cases, alcoholic stimulants should be withheld, for they act like poison and will often bring back delirium. Should stimulants be thought necessary (and it is not often really necessay) the carbonate of amonia, or the aromomatic spirits of amonia are preferable; or it may be proper, in some cases, to allow malt liquor. Opium in all forms, should be prohibited, until the system is relieved of its alcohol and even then I find it can generally be omitted; and when it can be, the patient recovers sooner and better. The patient is not expected to sleep well, but if the blood is renewed by its approprite nutriment, natural sleep will soon follow.

Occasionally, when, previous to the debauch which immediately caused the mania, a free use ot stimulants had been indulged in for some time, we have an exalted state of the nervous system, attended with hullucinations and sleeplessness which require special attention. Potent stimulants operate badly, and opium alone does not operate well, though in large doses sleep may be forced, though not without some risk, in some cases, to the brain; but equal parts of morphia and tart. antimony, given in small and repeated doses, will soon calm the nervous system and induce sleep without injury either to the brain or stomach. There is nothing that cools off the heated imagination in the cases like nauseating doses of tart. antimony, and opium in some form may be added, if it is thought necessary. The too general opinion that sleep is the all-important thing in this disease has led to fatal errors in treatment. Opium, given freely, as it often and very generally is, while the blood is charged with alcohol, produces a very unfavorable effect upon the nervous system, and tends to check the excretions, which are already diminished, and the patient, without being narcotized, often goes into a stupid state resembling the affects of uremic poison; and if about one-half (about the usual proportion), by the vigor of their constitutions, weather it, in spite of all the poisons imposed upon them they recover slowly, and their organs are left in a bad condition."

We make this lengthy extract from Prof. Stone's communication on this subject, because he makes the important distinction between delirium tremens and Mania a Potu, and in his usual clear manner points out the rational treatment in the two forms of the affection, these views we think should be more thoroughly impressed upon the profession, notwithstanding we are satisfied that the difference in the two forms of alcoholic poison is clear, the treatment of Dr Stone is the rational treatment yet the Mania a Potu that has occurred in this city for the last few years, has been remarkably fatal and all methods of treatment very unsatisfactory.

Therefore I determined to try the Tinct. Digitalis in large doses, recently, two. cases presented an opportunity. The first a man, the second a woman. A discription and treatment of one case will describe both in all essential particulars. Mr. ———, after a debauch of several weeks, and while yet stimulated to as great a degree as all the varieties of alcoholic drinks could produce; in attempting to light a segar, fell upon the floor in the most frightful convulsions raving, and foming at the mouth, and mutilating his lips, tongue, hands and arms with his teeth. In a half an hour, after this convulsion, he was furiously delirous, recognizing no one, muttering his imaginary fears and now ,and then making fearful struggles to escape from his bed, his face almost livid, eyes deeply injected and eye lids greatly swollen, pulse one hundred and sixty, weak and thread like.

In the interval of the convulsions and before my arrival, one half a grain of morphia had been administered to him. I immediately administered five grains of Ipecac and two grains Tarter Emetic, and repeated it every fifteen or twenty minutes, until he had ejected every thing from his stomach. This occupied several hours when his symptoms were not in the slightest improved. I then determined to watch the action of the digitalis and commenced by administering a large teaspoonful every half hour. The first dose improved his pulse, it diminished in frequency and increased in volume. The second dose lessened his ravings and made a more palpable improvent in his pulse. The third dose was increased one half, when all of his symptoms in one hour, were manifestly improving. I then increased the dose and lengthened the time of administration, in about seven hours from the taking of the first dose his pulse was full, slow and regular, his delirum had entirely vanished, and he was sleeping though interruptedly, now and then disturbed by his hallucinations. I then continued the Tinct. Digitalis in smaller doses, give a mercurial cathartic. His sleep became sound and tranquil, he awoke in about six hours, sane in mind and almost well in body. The only remaining vestage of disease, was extreme nervousness. I then prescribed Tinct. Valerian and compound tincture of cinchona in equal quantities. The second case was almost as severe as the first and was treated in like manner and resulted as favorably. If Digitalis acts by sedation, will not Veratria be a more efficient remedy in Mania a Potu?—*St. Joseph Medical and Surgical Journal.*

